# Effect of the ribotype 027 strain on *Clostridioides difficile* infection outcomes and recurrence in the Veterans Affairs healthcare system: a propensity scores matching study

**DOI:** 10.1186/s12879-026-13997-x

**Published:** 2026-07-10

**Authors:** Sandra Y. Silva, Brigid M. Wilson, Curtis J. Donskey

**Affiliations:** 1 Department of Population and Quantitative Health Science, Case Western Reserve University School of Medicine, Cleveland, OH USA; 2 Geriatric Research, Education, and Clinical Center, Louis Stokes Cleveland VA Medical Center, 10701 East Blvd, Cleveland, OH 44106 USA

**Keywords:** *Clostridioides difficile*, Ribotype 027, Mortality, Colectomy, Recurrence

## Abstract

**Background:**

*Clostridioides difficile* infection (CDI) caused by epidemic ribotype 027 strains has been associated with increased morbidity and mortality. However, many studies evaluating outcomes have been limited due to small sample size and inclusion of only one facility.

**Methods:**

Using a nationwide cohort of hospitalized patients in the Veterans Health System, we conducted a retrospective cohort study to examine the impact of infection with the 027 strain on severity of illness and recurrence in adults treated for CDI between October 2009 and September 2020. Cases of infection due to ribotype 027 were compared to control infections due to non-027 strains after balancing on patient characteristics using propensity score matching. Outcomes included recurrence of CDI, severe illness, and mortality at 14, 30, and 90 days after diagnosis.

**Results:**

In the unadjusted analysis, infection with ribotype 027 was associated with increased odds of recurrence and intensive care unit admission, severe CDI, colectomy, and death. For propensity score matching, 3709 cases of CDI due to ribotype 027 were successfully matched to 11,127 controls. After matching, infection with the ribotype 027 strain was only linked to increased risk of CDI recurrence (14.9% versus 10.5%, OR = 1.7, 95% CI, 1.55–1.86).

**Conclusion:**

After propensity score matching, we found that infection with the ribotype 027 strain was associated with recurrence of CDI, but not with increased severity of illness or mortality.

## Introduction

 In the early 2000s, the incidence and severity of *Clostridioides difficile* infection (CDI) increased in Europe and North America in conjunction with the emergence of the fluoroquinolone-resistant ribotype 027 strain [1, 2]. The ribotype 027 strain exhibits unique characteristics that could potentially contribute to an increase in morbidity and mortality, including increased sporulation, deletions in the *tcdC* gene that may result in increased toxin production, and binary toxin which has been proposed to increase adherence of *C. difficile* to the epithelium [3]. During the past decade, there have been significant reductions in the incidence of CDI and the proportion of cases due to the ribotype 027 strain in the United States and England which have been attributed in part to reductions in use of fluoroquinolones [4–6].

There is contradictory evidence regarding whether the ribotype 027 strain is associated with adverse outcomes. In some studies, the ribotype 027 strain has been associated with increases in CDI severity and recurrence [7–10], but other studies have not demonstrated significant increases in adverse outcomes or recurrence [11–16]. The conclusions from many of these studies have been limited due to small sample size and inclusion of only one facility. In a recent meta-analysis, it was concluded that the ribotype 027 strain was associated with unfavorable outcomes, but numerous studies were excluded due to discrepancies in the definition of the outcomes and the lack of reporting of important covariates [17]. Patients with CDI caused by the 027 strain tend to be older, with increased healthcare and antibiotic exposures, and more comorbidities [17]. These confounding differences may lead to biased estimates of the strain effect, making it difficult to determine if the 027 strain causes more severe disease versus afflicts a more vulnerable patient population. Propensity score analysis can minimize confounding bias by balancing the distribution of patient characteristics [18]. Here, we conducted a multicenter, retrospective study using propensity score matching to determine if ribotype 027 versus non-027 strains differ in CDI severity, mortality, and recurrence.

## Materials and methods

### Setting and study population

The Veterans Affairs (VA) system provides a unique opportunity to study the ribotype 027 *C. difficile* strain on a large scale because many VA hospitals use molecular tests that distinguish 027 from other strains and both individual patient and facility-level data can be analyzed [6]. The Veterans Administration’s Informatics and Computing Infrastructure was used to obtain data from the Corporate Data Warehouse, a central VA Healthcare System data repository containing administrative, clinical, laboratory, and pharmacy data linked using unique patient identifiers [6]. We generated a nationwide cohort of adult patients with 1 or more hospital admissions for any reason during the study period and a laboratory diagnosis of CDI in which the test result also classified the infecting strain as positive or negative for the 027 strain between October 1, 2009, and September 30, 2020. Testing for the 027-strain was performed using either the Xpert *C. difficile*/Epi Assay (Cepheid Sunnyvale, CA) or the Verigene *C. difficile* test (Luminex, Austin, TX). We excluded CDI patients who had positive CDI tests during the previous year because prior CDI is a risk factor for subsequent recurrence and our goal was to assess outcomes in patients with initial CDI.

We obtained patient-level data on demographics, comorbid illnesses, Braden Score, gastrointestinal procedures, laboratory test results, medications, and length of stay for inpatients. Charlson Comorbity Index Score (CCIS) was calculated. Prior antibiotic exposure was defined as one or more doses of systemic antibiotic therapy within 90 days prior to CDI diagnosis. Fluoroquinolones, macrolides, 2nd, 3rd, and 4th generation cephalosporins, clindamycin, carbapenems, and piperacillin-tazobactam were deemed high-risk antibiotics [19, 20].

Concomitant antibiotic use was defined as receiving two or more doses of high-risk antibiotics from the start of CDI treatment through 90 days after a positive CDI test [20]. CDI cases were classified as community-associated (CA-CDI) versus healthcare-associated (HA-CDI) based on standard surveillance definitions but using lab test events rather than symptoms to define CDI onset in relation to admission [21]. HA-CDI cases included both healthcare facility-onset CDI and community-onset, healthcare facility-associated CDI. Community-associated CDI included cases testing positive in the community or < 4 days from admission, provided that the positive test was > 12 weeks after the last discharge from a healthcare facility. For the purposes of the study, CDI recurrence was defined as a positive CDI test between 2 and 8 weeks after a previous positive test. Nasal screening results for methicillin-resistant *Staphylococcus aureus* (MRSA) within the previous 90 days were recorded if available.

### Outcomes

The primary outcomes were severe CDI (defined as white blood cell (WBC) count > 15,000 cells/mL and serum creatinine > 1.5 mg/dL within one day of the positive test [22]), intensive care unit (ICU) admission within seven days of a positive test, colectomy within 30 days of a positive CDI test, all-cause mortality within 14-, 30- and 90 days after a positive CDI test, and CDI recurrence.

### Statistical analysis

Continuous variables were summarized as mean and standard deviation; categorical variables were summarized as counts and percentages. Missing data were imputed using additive regression bootstrapping and predictive mean matching, with one hundred imputations conducted. Regression coefficients were pooled from the imputation dataset for the final propensity score model and a single set of propensity scores. To test whether imputation of missing data impacted the results, we conducted analyses including the complete case results.

We used propensity score matching analysis to adjust for confounding factors and balance baseline characteristics to assess the associations between CDI caused by the 027 strain and severity and recurrence. Propensity score matching was considered more appropriate than conventional multivariable logistic regression because patients infected with ribotype 027 strains differed substantially from those infected with non-027 strains in several baseline characteristics associated with CDI outcomes. While multivariable logistic regression can adjust for these factors, propensity score matching allows the creation of more comparable exposure groups by balancing the distribution of measured confounders prior to outcome analysis. Using multivariate logistic regression, we modeled the probability of acquiring CDI due to the 027 strain and used the estimated probability as a propensity score. Propensity scores were calculated as the conditional probabilities that a patient would have developed CDI due to the 027 strain given that patient’s measured baseline characteristics [23, 24].

We used “greedy nearest-neighbor” matching without replacement [18]. The matching algorithm first selected a ribotype 027 case and then selected a non-027 case with logit of the propensity score closest to the first case chosen. We used a 1:3 case: control ratio within a caliper width of 0.2 of the standard deviation of the logit of the propensity score. The balance of covariates before and after matching was assessed using the standardized mean difference (SMD), with absolute values of > 10% being the cutoff for poor match performance [18]. Conditional logistic regression analysis between the caliper-matched groups was performed to estimate the effect of 027 strain on the outcomes of interest. We applied a Bonferroni correction for multiple comparisons for the 7 outcomes included in the propensity score analysis.

We conducted a subgroup analysis by epidemiological classification. Healthcare-associated (HCA) and community-associated (CA) cases were matched with their respective controls. The same statistical approach described previously was used to compare ribotype 027 cases and controls for the subgroups. The analyses were performed using R version 4.1.1 statistical software (The R Foundation for Statistical Computing, Vienna, Austria) with the matching, survey, *twang*, and *cobalt* packages. Multiple imputation was done using the *Hmisc* package.

### Sensitivity analysis

Analyses based on inverse propensity score weighting were performed. Studies that used both propensity score matching and weighting have reported a low risk of bias [25]. For the average treatment effect in the treated group (ATT), the weights for the non-027 group were calculated as propensity score/(1—propensity), and the weights for subjects in the CDI 027 group were 1. ATT weighting with adjustment on the logit of the propensity score (double robust approach) was also performed.

## Results

### Study participants

Of 106,280 cases from 89 VA facilities testing positive for *C. difficile* with a simultaneous test for the 027 strain, 83,016 did not have a prior positive test within 1 year. Of these 83,016 CDI cases, 3,714 (4.5%) tested positive for the 027 strain and 79,302 (95.5%) tested negative. After propensity score matching, 3709 cases were included in the CDI 027 group and 11,127 cases in the non-027 group.

Overall, the proportion of missing data ranged from 2% to 18%, with similar rates in both the case and control groups. The average proportion of missing data across variables was 12.6%. The highest proportion of missing data was observed in the baseline characteristics, including the MRSA variable at 18%, followed by the Braden Scale at 17%, WBC count at 16%, creatinine at 13%, albumin at 11%, and body mass index at 3%. Outcomes variables had an overall missingness of 2%.

Table 1 provides a comparison of the baseline patient characteristics of CDI cases with infection due to the ribotype 027 strain versus non-027 strain before and after propensity score caliper matching. Before matching, in comparison to cases infected with non-027 strains, cases with CDI due to the ribotype 027 strain were more likely to be older, have higher comorbidity scores, lower serum albumin, higher peripheral WBC count, higher serum creatinine, lower Braden score, more frequent MRSA colonization, more frequent exposure to high-risk antibiotics, and higher likelihood of having healthcare-associated CDI. After matching, the imbalance between the 027 and non-027 groups was greatly reduced, with standardized mean differences of less than 10%, suggesting a covariate balance between the groups.

**Table 1:** Comparison of baseline patient characteristics before versus after propensity score caliper matching

	Before matching			After 1: 3 matching		
Characteristic	All CDI-027 cases (*n* = 3,714)	All non-027 cases (*n* = 79,302)	SMD (%)	Matched CDI-027 cases (*n* = 3,709)	Matched non-027 cases (*n* = 11127)	SMD (%)
Gender Male (%)	3544 (95.4)	74,510 (94.0)	7.01	3539 (95.4)	10,650 (95.7)	-1.41
Age, years (mean (SD))	71.51 (12.70)	68.10 (13.46)	26.82	71.50 (12.71)	71.64 (12.44)	-1.14
BMI (mean (SD))	27.19 (8.18)	27.56 (8.20)	-4.61	27.18 (8.07)	27.10 (7.81)	1.02
CCIS (mean (SD))	4.25 (3.25)	3.88 (3.21)	11.5	4.25 (3.25)	4.21 (3.22)	1.4
Braden Scale (mean (SD))	16.45 (2.92)	16.87 (2.78)	-14.99	16.45 (2.77)	16.42 (2.78)	1
Inflammatory Bowel Disease (%)	62 (1.7)	2795 (3.5)	-14.47	62 (1.7)	162 (1.5)	1.68
Diabetes Mellitus (%)	1598 (43.0)	30,850 (38.9)	8.33	1593 (42.9)	4756 (42.7)	0.42
Cancer (%)	985 (26.5)	21,377 (27.0)	0.99	983 (26.5)	2907 (26.1)	0.86
Cirrhosis (%)	259 (7.0)	5184 (6.5)	1.71	259 (7.0)	729 (6.6)	1.69
Gastrointestinal procedure	78 (2.1)	2107 (2.7)	-3.88	78 (2.1)	235 (2.1)	-0.06
Nasal colonization MRSA (%)	588 (20.3)	8798 (15.1)	12.98	585 (15.8)	1772 (15.9)	-0.42
Serum creatinine, mg/dL (mean (SD))	1.71 (1.59)	1.66 (1.71)	3.93	1.70 (1.50)	1.69 (1.55)	0.61
Peripheral WBC count, k/mm^3^ (mean (SD))	12.84 (8.40)	11.08 (7.16)	19.81	12.49 (7.42)	12.36 (8.31)	1.62
Serum albumin, g/dL (mean (SD))	3.00 (0.70)	3.11 (0.77)	-16.91	3.01 (0.67)	3.01 (0.71)	0.19
Any-high risk antibiotic *	2468 (66.5)	47,335 (59.7)		2463 (66.4)	7374 (66.3)	
Proton pump inhibitors (%)	2126 (57.2)	47,070 (59.4)	-5.36	2123 (57.2)	6612 (59.4)	0.25
Fluoroquinolones (%)	1265 (34.1)	23,888 (30.1)	8.31	1262 (34.0)	3710 (33.3)	1.44
Clindamycin (%)	215 (5.8)	4095 (5.2)	2.68	215 (5.8)	661 (5.9)	-0.62
Macrolides (%)	458 (12.3)	6072 (7.7)	14.21	454 (12.2)	1362 (12.2)	0
Second-generation cephalosporins (%)	83 (2.2)	2153 (2.7)	-3.25	83 (2.2)	230 (2.1)	1.15
Third-generation cephalosporins (%)	860 (23.2)	15,138 (19.1)	9.64	857 (23.1)	2510 (22.6)	1.3
Fourth-generation cephalosporins (%)	445 (12.0)	6665 (8.4)	11.01	442 (11.9)	1311 (11.8)	0.42
Piperacillin-Tazobactam (%)	1101 (29.6)	21,797 (27.5)	4.73	1098 (29.6)	3363 (30.2)	-1.36
Carbapenem (%)	298 (8.0)	6097 (7.7)	1.23	298 (8.0)	891 (8.0)	0.01
Concomitant high risk antibiotic use (%) *	1617 (43.5)	33,888 (42.7)		1615 (43.5)	5171 (46.5)	
Facility Complexity						
1	3036 (81.7)	64,468 (81.3)	1.17	3033 (81.8)	9117 (81.9)	-0.42
2	361 (9.7)	6714 (8.5)	4.23	359 (9.7)	1027 (9.2)	1.52
3	211 (5.7)	4260 (5.4)	1.34	211 (5.7)	651 (5.9)	-0.7
Other	106 (2.9)	3860 (4.9)		106 (2.9)	332 (3.0)	
Epidemiological Classification						
Community Associated	1064 (28.6)	28,044 (35.4)	-14.85	1064 (28.7)	3119 (28.0)	1.45
Healthcare Associated	2468 (66.5)	46,794 (59.0)	15.76	2463 (66.4)	7460 (67.0)	-1.35
Undetermined	182 (4.9)	4464 (5.6)		182 (4.9)	548 (4.9)	

Abbreviations: CDI, *Clostridioides difficile* infection; SMD, standardized mean difference; SD, standard deviation; BMI, body mass index; CCIS, Charlson Comorbidity Index Score; MRSA, methicillin-resistant *Staphylococcus aureus*; WBC, white blood cell

High-risk antibiotics included fluoroquinolones, macrolides, 3rd generation cephalosporins, clindamycin, and piperacillin-tazobactam

* These composite variables were not included in the propensity score model because the individual antibiotic classes were included and inclusion of the composite variables would have introduced redundancy and potential multicollinearity into the propensity model

### Outcomes of infection due to the ribotype 027 strain versus other strains

Of the 83,016 *C. difficile* cases, 8138 (9.8%) had a CDI recurrence, and 23,897 (28.8%) had 1 or more of the other adverse outcomes. The odds ratios with and without propensity score matching are shown in Table 2. Propensity-matched subjects with CDI due to 027 strains had significantly higher odds of CDI recurrence (OR = 1.7; 95% CI, 1.55–1.86). Conversely, the odds of death within 14 days (OR = 1.02; 95% CI, 0.89, 1.18), 30 days (OR = 1.06; 95% CI, 0.95, 1.19), and 90 days (OR = 1.04; 95% CI, 0.95, 1.14) of a positive CDI test, ICU admission (OR = 1.12; 95% CI, 0.99, 1.23), severe disease (OR = 1.14; 95% CI, 0.97, 1.34), and colectomy (OR = 1.58; 95% CI, 0.95–2.62) were not statistically different between CDI cases caused by 027 versus non-027 strains. The results were similar when the propensity-matched analysis was completed using the complete case results (i.e., without imputation of missing data) comparing patients with CDI due to 027 strains versus non-027 strains: CDI recurrence (OR = 1.5; 95% CI, 1.27–1.77; *P* < 0.001), odds of death within 30 days (OR = 1.09, 95% CI 0.91–1.30; *P* = 0.34), ICU admission (OR 1.05, 95% CI 0.90–1.23; *P* = 0.53), and severe disease (OR = 1.0, 95% CI 0.79–1.27; *P* = 1.00).

**Table 2:** Propensity score (PS) matched odds ratios and 95% confidence intervals (CI) for adverse CDI outcomes and CDI recurrence in patients with CDI due to the ribotype 027 strain compared with non-027 strains

Outcome	Full studied population *n* (%)		Unadjusted	Propensity-matched groups *n* (%)		PS caliper matching	
	CDI 027 cases *n* = 3,714	non-CDI 027 cases *n* = 79,302	OR (95% CI)	CDI 027 cases *n* = 3,709	non-CDI 027 cases *n* = 11,127	OR (95% CI)	*p* value
All-cause mortality 14 days	277 (7.5)	4274 (5.4)	1.42 (1.25–1.61)	275 (7.4)	809 (7.3)	1.02 (0.89–1.18)	0.77
All-cause mortality 30 days	482 (13.0)	7465 (9.4)	1.44 (1.30–1.59)	479 (12.9)	1370 (12.3)	1.06 (0.95–1.19)	0.33
All-cause mortality 90 days	840 (22.6)	13,593 (17.1)	1.41 (1.31–1.53)	836 (22.5)	2437 (21.9)	1.04 (0.95–1.14)	0.4
ICU Admission	546 (18.0)	9872 (15.3)	1.21 (1.10–1.33)	545 (18)	1529 (16.8)	1.12 (0.99–1.23)	0.06
Severe CDI	230 (6.2)	3064 (3.9)	1.64 (1.43–1.89)	227 (6.1)	608 (5.5)	1.14 (0.97–1.34)	0.12
Colectomy	23 (0.6)	319 (0.4)	1.54 (1.01–2.36)	23 (0.6)	44 (0.4)	1.58 (0.95–2.62)	0.08
Recurrence	551 (14.8)	7587 (9.6)	1.65 (1.50–1.81)	551 (14.9)	1165 (10.5)	1.7 (1.55–1.86)	< 0.001

Abbreviations: CDI, *Clostridioides difficile* infection; OR, odds ratio; ICU, intensive care unit

Severe CDI was defined as white blood cell (WBC) count > 15,000 cells/mL or serum creatinine > 1.5 mg/dL within one day of the positive test [19]

In the analysis stratified by epidemiological classification, CA CDI accounted for 35.1% of CDI cases, with a higher representation in the non-027 group (35.4%) versus the ribotype 027 group (28.6%). Following the calculation of propensity scores and the matching of ribotype 027 with non 027 cases across each classification, the odds of recurrence was significantly higher in the matched ribotype 027 versus the non-027 cases. The effect size of the recurrence odds ratios was greater for patients with CA CDI **(**Table 3**)**.

**Table 3:** Propensity Score (PS) matched Odds ratio and 95% confidence intervals (CI) for CDI outcomes in patients with CDI due to 027 strain compared with non-027 strains, stratified by healthcare-associated (a) versus community-associated (b) CDI

**a. Health Care Associated (HA) CDI**							
**Outcome**	**Full HA-CDI population n(%)**	**Unadjusted**	**Propensity-matched groups n(%)**	**PS caliper matching**		**PS caliper matching**	
	**CDI 027 cases n=2,468**	**non-CDI 027 cases n=46,794**	**OR (95% CI)**	**CDI 027 cases n=2465**	**non-CDI 027 cases n=7395**	**OR (95% CI)**	***p* value**
All-cause mortality 14 days	213 (8.6)	3462 (7.4)	1.18 (1.02-1.37)	212 (8.6)	648 (8.8)	0.98 (0.83-1.16)	0.8
All-cause mortality 30 days	379 (15.4)	5989 (12.8)	1.24 (1.11-1.39)	378 (15.3)	1147 (15.5)	0.99 (0.87-1.12)	0.83
All-cause mortality 90 days	664 (26.9)	10,697 (22.9)	1.24 (1.13-1.36)	663 (26.9)	1981 (26.8)	1.01 (0.90-1.12)	0.91
ICU Admission *	372 (18.4)	6593 (16.9)	1.11 (0.99-1.24)	371 (18.4)	1059 (17.5)	1.06 (0.93-1.21)	0.36
Severe disease	171 (6.9)	2362 (5.0)	1.40 (1.19-1.64)	169 (6.9)	477 (6.5)	1.07 (0.89-1.29)	0.47
Colectomy	19 (0.8)	227 (0.5)	1.59 (0.99-2.55)	19 (0.8)	40 (0.5)	1.42 (0.82-2.44)	0.21
Recurrence	363 (14.7)	4923 (10.5)	1.47 (1.31-1.65)	363 (14.7)	820 (11.1)	1.38 (1.21-1.58)	<0.001
**b. Community Associated (CA) CDI**							
**Outcome**	**Full CA-CDI population n (%)**	**Unadjusted**	**Propensity-matched groups n (%)**	**PS caliper matching**		**PS caliper matching**	
	**CDI 027 cases *n*=1**,**064**	**non-CDI 027 cases n= 28,044**	**OR (95% CI)**	**CDI 027 cases n= 1, 063**	**non-CDI 027 cases n= 3, 189**	**OR (95% CI)**	***p* value**
All-cause mortality 14 days	63 (5.9)	794 (2.8)	2.16 (1.66-2.81)	63 (5.9)	147 (4.6)	1.31 (0.97-1.78)	0.08
All-cause mortality 30 days	100 (9.4)	1442 (5.1)	1.91 (1.55-2.37)	99 (9.3)	258 (8.1)	1.18 (0.92-1.51)	0.2
All-cause mortality 90 days	169 (15.9)	2816 (10.0)	1.69 (1.43-2.00)	168 (15.8)	483 (15.1)	1.06 (0.86-1.30)	0.58
ICU Admission *	171 (19.4)	3241 (14.2)	1.44 (1.22-1.71)	171 (19.4)	429 (16.2)	1.25 (1.02-1.51)	0.03
Severe disease	59 (5.5)	696 (2.5)	2.3 (1.76-3.03)	59 (5.6)	138 (4.3)	1.33 (0.96-1.85))	0.09
Colectomy	4 (0.4)	92 (0.3)	1.15 (0.42-3.13)	4 (0.4)	8 (0.3)	1.5 (0.45-4.98)	0.51
Recurrence	163 (15.3)	2315 (8.3)	2.01 (1.69-2.39)	163 (15.3)	258 (8.1)	2.04 (1.66-2.52)	<0.001

Abbreviations: CDI, *Clostridioides difficile* infection; OR, odds ratio; ICU, intensive care unit

Severe CDI was defined as white blood cell (WBC) count > 15,000 cells/mL or serum creatinine > 1.5 mg/dL within one day of the positive test [19]

### Sensitivity analysis

Table 4 summarizes the estimates of the studied outcomes across different propensity score methods. Consistent odds ratios were observed across all models that incorporated propensity matching, weighting, or both.

**Table 4:** Comparison of unadjusted and propensity score estimates on outcomes

Outcome	Method	Odds Ratio (95% CI)
All-Cause Mortality 14 days	Unadjusted	1.42 (1.25–1.61)
	PS caliper matching	1.02 (0.89–1.18)
	PS ATT weighting	1.03 (0.91–1.17)
	Double robust	1.04 (0.92–1.19)
All-Cause Mortality 30 days	Unadjusted	1.44 (1.30–1.59)
	PS caliper matching	1.06 (0.95–1.19)
	PS ATT weighting	1.05 (0.95–1.16)
	Double robust	1.06 (0.96–1.18)
All-Cause Mortality 90 days	Unadjusted	1.41 (1.31–1.53)
	PS caliper matching	1.04 (0.95–1.14)
	PS ATT weighting	1.04 (0.96–1.13)
	Double robust	1.05 (0.97–1.14)
ICU Admission	Unadjusted	1.21 (1.10–1.33)
	PS caliper matching	1.12 (0.99–1.23)
	PS ATT weighting	1.09 (0.99–1.20)
	Double robust	1.1 (0.99–1.21)
Severe CDI	Unadjusted	1.64 (1.43–1.89)
	PS caliper matching	1.14 (0.97–1.34)
	PS ATT weighting	1.07 (0.93–1.23)
	Double robust	1.10 (0.95–1.27)
Colectomy	Unadjusted	1.54 (1.01–2.36)
	PS caliper matching	1.58 (0.95–2.62)
	PS ATT weighting	1.35 (0.88–2.08)
	Double robust	1.35 (0.88–2.08)
Recurrence	Unadjusted	1.65 (1.50–1.81)
	PS caliper matching	1.7 (1.55–1.86)
	PS ATT weighting	1.54 (1.41–1.70)
	Double robust	1.55 (1.41–1.70)

Abbreviations: PS, propensity score; ATT, average treatment effect in the treated group; CDI, *Clostridioides difficile* infection; OR, odds ratio; ICU, intensive care unit

Severe CDI was defined as white blood cell (WBC) count > 15,000 cells/mL or serum creatinine > 1.5 mg/dL within one day of the positive test [19]

For the ATT, the weights for the non-027 group were propensity score/(1—propensity), and the weights for subjects in the CDI 027 group were 1. Double robust indicates ATT weighting with adjustment on the logit of the propensity score

## Discussion

Infection with the ribotype 027 strain has been associated with severe CDI and adverse outcomes, including recurrence [17], but several reports have not confirmed an independent association between the ribotype 027 strain and adverse outcomes [11–16]. Here, we used propensity score analysis to compare CDI outcomes and recurrence in a large cohort of patients infected with ribotype 027 versus non-027 strains. At baseline, patients infected with ribotype 027 strains were significantly older with higher Charlson comorbidity scores and greater exposure to high-risk antibiotics. In the unadjusted analysis, infection with ribotype 027 was associated with increased odds of recurrence, ICU admission, severe CDI, colectomy, and death. However, after propensity score matching, infection with the ribotype 027 strain was only significantly linked to increased risk of recurrence (14.9% versus 10.5%). These findings suggest that confounding factors such as increased age and comorbidities may contribute to increased severity of infection due to ribotype 027 strains, whereas strain characteristics might contribute to an increase in CDI recurrence.

The specific characteristics of the ribotype 027 strain that might result in an increased risk for recurrence are uncertain. One consideration is that infection with ribotype 027 strains has been associated with an increased likelihood of having positive toxin results on enzyme immunoassay testing [26, 27]. Positive stool enzyme immunoassay results have been associated with increased likelihood of developing recurrence of CDI [28]. However, ribotype 027 strains may vary considerably in toxin production, and 027 isolates with low toxin production have been shown to exhibit robust virulence and heightened colonization and persistence in rodent models [29]. Thus, there is a need for additional studies to examine factors that might contribute to increased recurrence in patients infected with ribotype 027 strains.

Some clinical assays can provide presumptive identification of 027 strains [6]. Although this information is not typically used clinically, it is plausible that it could be used to target infection control or antimicrobial stewardship interventions for CDI patients infected with 027 strains at high risk for adverse outcomes and recurrence. For example, Widmer et al. [30] discontinued use of contact precautions for CDI patients infected with ribotypes other than 027 or 078 unless incontinent and noted no increase in CDI rates or outbreaks over a 10-year period.

Our study has several strengths. We included a large cohort of patients from multiple hospitals in all regions of the United States over a 13-year period. We used propensity score matching which has potential advantages over traditional regression analysis when estimating causal effects [31]. After propensity score matching, the imbalance between the 027 and non-027 groups was minimal, with standardized mean differences of less than 10%.

Our study also has some limitations. Although our propensity model includes characteristics that could affect CDI severity, we cannot exclude the possibility that differences between the groups may still exist, resulting in biased estimates. Data extraction was performed electronically rather than through review of individual medical records. We cannot exclude the possibility of some errors in data extraction or missing information (e.g., transfer of patients to non-VA facilities). We used positive test results to identify CDI cases and recurrences as we were unable to assess symptoms. It is possible that some recurrences represented colonization rather than CDI. Our results do not provide a clear explanation for why the ribotype 027 strain more often infects older adults with higher Charlson comorbidity scores and greater exposure to high-risk antibiotics. One consideration is that ribotype 027 strains are more often associated with healthcare-associated infections, possibly due to an increased propensity to spread and cause infections in healthcare facilities [1, 2, 5, 32]. The Veteran study population includes mostly males and may not be generalizable to all populations, including those outside the United States. It is possible that some cases of recurrence may have been missed if they were diagnosed outside the VA Healthcare System. Finally, the cohort included only patients with 1 or more hospitalizations for any reason during the study period and therefore may not have included some cases of community-associated CDI.

In conclusion, after adjusting for variables likely to predispose patients to both the ribotype 027 strain and adverse infection outcomes, infection with the ribotype 027 strain was associated with recurrence of CDI but not with increased severity of illness or mortality. These results suggest that the ribotype 027 strain may be associated with more severe disease because it infects vulnerable patients rather than due to increased virulence. The substantial increase in risk of recurrence associated with ribotype 027 has important implications for the care of CDI patients infected with this strain. Assays that identify the 027 strain are commonly used, and the information can be made readily available to clinicians at the time of diagnosis. Treatment algorithms could potentially be modified to provide guidance that patients infected with the 027 strain should be treated with agents that are less likely to be associated with recurrence (e.g., fidaxomicin). Enhanced infection control measures could also potentially be implemented to reduce the risk for recurrence (e.g., patient hand hygiene and bathing, cleaning surfaces with bleach).

## Data Availability

The datasets used and/or analyzed during the current study are available from the corresponding author on reasonable request.

## Abbreviations

CDI: *Clostridioides difficile*
VA: Veterans Affairs
WBC: White blood cell
MRSA: Methicillin-resistant *Staphylococcus aureus*
SMD: Standardized mean difference
HCA: Healthcare-associated
CA: Community-associated
OR: Dds ratio
CI: Confidence interval
SD: Standard deviation
BMI: Body mass index
CCIS: Charlson Comorbidity Index Score
ATT: Average treatment effect in the treated group
PS: Propensity score
